# Secondhand tobacco smoke exposure and heart rate variability and inflammation among non-smoking construction workers: a repeated measures study

**DOI:** 10.1186/1476-069X-12-83

**Published:** 2013-10-02

**Authors:** Jinming Zhang, Shona C Fang, Murray A Mittleman, David C Christiani, Jennifer M Cavallari

**Affiliations:** 1 Department of Environmental Health, Harvard School of Public Health, Boston, Massachusetts, USA; 2 Division of Occupational and Environmental Medicine, University of Connecticut Health Center, Farmington, Connecticut, USA; 3 Department of Epidemiology, Harvard School of Public Health, Boston, MA, USA; 4 Department of Medicine, Massachusetts General Hospital/Harvard Medical School, Boston, MA, USA; 5 Harvard Medical School, Cardiovascular Epidemiology Research Unit, Beth Israel Deaconess Medical Center, Boston, MA, USA; 6 New England Research Institutes, Inc, Watertown, Massachusetts, USA

**Keywords:** Secondhand smoke, Tobacco, Autonomic nervous system, Heart rate variability, Inflammatory marker

## Abstract

**Background:**

Although it has been well recognized that exposure to secondhand tobacco smoke (SHS) is associated with cardiovascular mortality, the mechanisms and time course by which SHS exposure may lead to cardiovascular effects are still being explored.

**Methods:**

Non-smoking workers were recruited from a local union and monitored inside a union hall while exposed to SHS over approximately 6 hours. Participants were fitted with a continuous electrocardiographic monitor upon enrollment which was removed at the end of a 24-hr monitoring period. A repeated measures study design was used where resting ECGs and blood samples were taken from individuals before SHS exposure (baseline), immediately following SHS exposure (post) and the morning following SHS exposure (next-morning).

Inflammatory markers, including high sensitivity C-reactive protein (CRP) and white blood cell count (WBC) were analyzed. Heart rate variability (HRV) was analyzed from the ECG recordings in time (SDNN, rMSSD) and frequency (LF, HF) domain parameters over 5-minute periods. SHS exposure was quantified using a personal fine particulate matter (PM_2.5_) monitor.

Linear mixed effects regression models were used to examine within-person changes in inflammatory and HRV parameters across the 3 time periods. Exposure-response relationships with PM_2.5_ were examined using mixed effects models. All models were adjusted for age, BMI and circadian variation.

**Results:**

A total of 32 male non-smokers were monitored between June 2010 and June 2012. The mean PM_2.5_ from SHS exposure was 132 μg/m^3^. Immediately following SHS exposure, a 100 μg/m^3^ increase in PM_2.5_ was associated with declines in HRV (7.8% [standard error (SE) =3%] SDNN, 8.0% (SE = 3.9%) rMSSD, 17.2% (SE = 6.3%) LF, 29.0% (SE = 10.1%) HF) and increases in WBC count 0.42 (SE = 0.14) k/μl. Eighteen hours following SHS exposure, a 100 μg/m^3^ increase in PM_2.5_ was associated with 24.2% higher CRP levels.

**Conclusions:**

Our study suggest that short-term SHS exposure is associated with significantly lower HRV and higher levels of inflammatory markers. Exposure-associated declines in HRV were observed immediately following exposure while higher levels of CRP were not observed until 18 hours following exposure. Cardiovascular autonomic and inflammation responses may contribute to the pathophysiologic pathways that link SHS exposure with adverse cardiovascular outcomes.

## Background

Cardiovascular disease is one of the leading causes of death worldwide and there is sufficient scientific evidence that secondhand smoke (SHS) exposures cause morbidity and mortality from cardiovascular diseases (CVD) [1-6]. The 2006 report from the US Surgeon General estimates a 25 to 30% increase in risk from coronary heart disease from SHS exposure [7]. Unlike chronic active smoking where mechanisms of cumulative atherogenesis affect CVD risk, a synthesis of epidemiological and toxicological research suggests that SHS exposures may be an acute threat to the cardiovascular system with effects characterized by rapid onset [8]. In fact, many studies have indicated that short-term exposure to SHS may act as a trigger of acute clinical cardiovascular responses acting through multiple mechanisms including alteration of cardiovascular autonomic system and inflammation [9-12]. Changes in cardiovascular autonomic control are often assessed using measures of heart rate variability (HRV) which is the beat-to-beat variability of the R-R interval of successive normal beats on an electrocardiogram (ECG). Lower levels of HRV are strong predictors of mortality [13] especially in cardio-compromised patients [14]. Autonomic nervous system influenced changes in HRV may increase the likelihood of sudden cardiac death [15]. White blood cell count (WBC) and C-reactive protein (CRP) are markers of inflammation that have been associated with an increased incident of coronary heart disease.

Due to significant advances in state laws banning cigarette smoking in indoor public spaces, smoking rates continue to decline in the general population [16]. However, the smoking rates among blue-collar occupations, such as construction workers, remain high. Studies have indicated that the prevalence of smoking, especially heavy smoking, was significantly higher among construction workers comparing with the general population [17-19]. For example, the National Health Interview Survey found that construction workers maintain high smoking rates with 39% of construction workers smoking in 1987–2004 [17]. Due to the nature of construction work with a mix of indoor and outdoor work being performed, “indoor” smoking bans do not always apply, leaving a large population at risk for SHS exposures.

We conducted a repeated measures study among a panel of non-smoking boilermaker construction workers. In this study, we attempted to clarify the relationship between acute SHS exposure and cardiovascular effects. We hypothesized that acute SHS exposure, which was quantified by fine particulate matter (PM_2.5_), would be associated with lower HRV parameters and higher levels of inflammatory markers.

## Methods

### Study population

Boilermaker construction workers were recruited from members of a local boilermaker union in Quincy, Massachusetts, USA. Participants were invited to participate in the study through letters sent by union leadership. Boilermaker construction workers are trained to weld on round vessels or boilers located in power plants. As part of a larger study, boilermaker construction workers were monitored to understand the health effects of their workplace exposures including welding fume and secondhand smoke (SHS). As part of the current study, workers who were currently non-smokers were invited to participate. There were 17 participants who had never smoked, 13 participants who had quit smoking for at least 3 years before the study and two participants quit smoking 6 months before the study. The participants in the current study included 32 non-smoking workers, recruited over four site visits in June 2010, January and June in 2011 and June 2012. The workers were allowed to participate multiple times during the four sampling periods. Participants were monitored at a union welding school where welders learn and practice welding techniques on days when no welding occurred. Participants were monitored over 6 hours when they performed book work as part of the apprentice training program, read, played card games and completed questionnaires. During the SHS exposure days, participants stayed in the enclosed welding room where smoking welders were allowed to smoke. Therefore, it was hypothesized that participants were only exposed to SHS derived particulate matter. The resting ECGs and blood samples were collected from each participant at three time points: before SHS exposure (baseline), following SHS exposure (post) and the morning following SHS exposure (next-morning). Participants also completed self-administered questionnaires including weight, height, age, race, the last day they performed welding, smoking status, medical history and medication use. All measurements were made in the same location under the same setup. The study was approved by the Harvard School of Public Health Institutional Review Board and written informed consent was obtained from all participants.

### Electrocardiographic monitoring

Participants were fitted with a standard ambulatory electrocardiogram (ECG) Holter monitor. The ECG has the advantage of being a non-invasive method of monitoring the electrical activity of the heart. The Holter monitor continuously records electrical signals from the heart and the data collected from the ECG can be used to assess changes in heart rate variability, a measure of cardiovascular autonomic control. During the site visit, each participant rested for 12-minutes, with the exception of June 2010 when participants rested for 7 minutes. During the resting period, participants were asked to remain quiet in a seated position without talking or eating. The resting period was performed for each participant at baseline, post-exposure, and the next morning. The ECG records were analyzed by trained technicians, blind to exposures, in the Cardiovascular Epidemiology Research Unit of Beth Israel Deaconess Medical Center. The ECG records were analyzed to quantify HRV in the time and frequency domains including the square root of the mean of the sum of the squared differences between adjacent normal to normal intervals (rMSSD), the standard deviation of all NN intervals over the entire period (SDNN), the low-frequency power (LF) and the high-frequency power (HF). The continuous HRV monitoring data were summarized every 5-minute and the 5-minute values coordinating with the resting ECG were selected for analyses. For 7-min recordings, the first two minutes of the resting data were discarded to allow for acclimation. For 12-min recordings, two 5-minute values were summarized for 3th-7th and 8th-12th minute intervals after excluding the first two minutes. In total, there were 284 observations of 5-minute values from all participants.

### Markers of inflammation sampling and analyses

Blood samples were drawn at baseline, post-exposure and next-morning by a phlebotomist using standard clinical procedures. The blood samples were immediately sent to Quest Diagnostics, a CLIA-certified clinical laboratory, for analysis. Inflammatory markers including a complete blood count with platelets were analyzed to determine white blood cell count, platelets, neutrophils and other inflammatory cells. The blood was also analyzed for C-reactive protein (CRP), a marker of inflammation and predictor of coronary heart disease. We obtained 144 observations for CRP and 150 observations for whole blood cell counts from the research population.

### Acute secondhand smoke exposure assessment

SHS exposures were quantified by particles ≤2.5 μm in aerodynamic diameter (PM_2.5_). PM_2.5_ has the ability to travel deep into the alveolar regions of the lungs where it can exert local and systemic pulmonary and cardiovascular health effects [20]. A Sidepak™ Aerosol Monitor (TSI, Inc., St. Paul, MN) was used to obtain minute-to-minute average, personal breathing zone PM_2.5_ concentrations. The personal PM_2.5_ monitor received yearly calibrations. The internal PM_2.5_ impactor, which was cleaned daily, was used at a 1.7 L/min flow rate which was confirmed prior to each use. The PM_2.5_ measurements were averaged over the exposure period. The Sidepak™ has been successfully used to monitor short-term SHS exposures [21].

### Statistical analysis

We used linear mixed regression models to investigate changes in the post-exposure and the next-morning HRV and levels of inflammatory markers as compared to baseline. The HRV parameters, CRP and lymphocyte concentrations were log-transformed due to the skewed distribution of model residuals. In this study, some baseline samples were taken in the morning while others were taken in the afternoon. As HRV and markers of inflammation were known to exhibit a circadian pattern [22,23], we controlled for the circadian variation by using an indicator-time when baseline samples were taken in the model: in the morning or the afternoon. Separate models were constructed for the each outcome and for either post-exposure or next-morning time period. All models were adjusted for time (baseline, post-exposure, or next-morning), BMI, age and circadian variation.The regression coefficients, standard errors and p-values were estimated from the models.

Linear mixed regression models were also used to examine the relationship between HRV or inflammatory markers and PM_2.5_ concentrations (PM model). We added random intercepts for each model and the compound symmetry correlation structure was considered. In these models, baseline measurements were included since the SHS exposure occurred after the baseline measurements were made. The models were also adjusted for BMI, age and time when baseline samples were collected to account for circadian variation. Once again, separate models were constructed for the each outcome and for either post-exposure or next-morning time period. In a sensitivity analyses, 2 participants who reported taking statins or beta-blockers were excluded since HRV and markers of inflammation may be affected by these classes of medications. All statistical analyses were performed with SAS v 9.3 (SAS Institute Inc, NC). We set the statistical significance with a two-sided α = 0.05.

## Results

Demographic descriptions of the research population are shown in Table 1. Among 32 male boilermaker construction workers, the age ranged from 21 to 71 years (mean = 44.5). Most of the participants were relatively healthy, but 2 subjects reported past cardiovascular history and statin or beta-blockers usage. Within the 4 sampling periods, 18 subjects participated once, 6 participated two times, 6 participated three times and 2 participated all four times. All participants reported that they had not performed welding for at least a week before the monitoring day. In total, there were 56 observations from the 32 participants. Characteristics of PM_2.5_ measurements are summarized in Table 2. The average PM_2.5_ measurement during the SHS exposure was 132.3 μg/m^3^(with a range from 6.5-506.9 μg/m^3^). The time weighted average (TWA) PM_2.5_ exposure after adjusting for different sampling hours among participants was 103.8 μg/m^3^(with a range from 4.4 to 380.2 μg/m^3^).

**Table 1:** Participant demographics

**Characteristics**	**N(%) or Mean ± SD**
Male	32(100)
Age(years)	44.5 ± 13.3
Range	21.2-71.2
BMI(kg/m^2^)	28 ±4
Race	
White	26(81.3)
Black	3(9.4)
Hispanic	2(6.3)
Asian	1(3.0)
Cardiovascular(CV) History	
Hypertensive	2(6.3)
High Cholesterol	2(6.3)
Arrhythmia	2(6.3)
Statin or Beta-blocker Use	2(6.3)

**Table 2:** **Characteristics of PM**_**2.5 **_**exposure**

**Characteristic**	**Mean**	**SD**	**Q25**	**Q50**	**Q75**
PM_2.5_ Exposure(μg/m^3^)	132.3	124.1	42.9	79.8	177.4
Time weighted average(μg/m^3^)	103.8	100.2	32.2	59.9	133.1
PM_2.5_ measurement time(min)	349	18.6	347	360	360

The mean levels of the HRV parameters at baseline, post SHS exposure and the next morning are summarized in Table 3. After SHS exposure, both SDNN (53.1 msec vs. 47.9 msec) and LF (1164 msec^2^ vs. 961 msec^2^) were significantly lower than baseline (i.e. pre-exposure) values. SDNN and LF then returned to baseline levels the next morning and no significant differences were observed between the next-morning and baseline values. We did not observe significant differences in rMSSD and HF at either post SHS exposure or the next morning.

**Table 3:** **HRV parameters and inflammatory markers by time (Mean ± SD)**^**a,b**^

**HRV measures**	**Baseline**	**Post**	***p *** **Value**^**c**^	**Next morning**	***p *** **Value**^**d**^
Heart rate(bpm)	69.0(14.0)	71.6(12.5)	0.061	68.4(10.7)	0.916
SDNN(msec)	53.1(25.4)	47.9(20.1)	0.026	51.8(24.1)	0.149
rMSSD(msec)	28.6(17.9)	28.2(20.0)	0.429	28.5(19.3)	0.594
LF(msec^2^)	1164(1366)	961(1020)	0.039	1101(1309)	0.115
HF(msec^2^)	431(680)	518(996)	0.644	440(753)	0.754
CRP(mg/L)	1.89(2.03)	2.04(1.80)	0.371	2.53(2.40)	0.036
Lymphocytes(k/μl)	2.06(5.12)	2.16(5.61)	0.079	2.02(5.65)	0.451
Red blood cells(M/μl)	4.91(0.38)	4.83(0.33)	0.020	4.91(0.39)	0.746
White blood cells(k/μl)	6.80(1.57)	7.33(1.91)	0.010	7.14(2.35)	0.145
Neutrophils(k/μl)	3.96(1.23)	4.34(1.51)	0.031	4.33(1.98)	0.031
Monocytes(k/μl)	0.55(0.16)	0.59(0.18)	0.094	0.56(0.18)	0.761
Basophils(k/dl)	0.03(0.01)	0.03(0.01)	0.591	0.03(0.01)	0.577
Eosinophils(k/μl)	0.25(0.20)	0.24(0.23)	0.781	0.25(0.27)	0.424
Platelets(k/μl)	217(35)	222(36)	0.026	218(39)	0.577

^a^Exposure to SHS occurred from baseline to post.

^b^Unadjusted Means(SDs).

^c^Comparison between baseline and post using linear mixed regression models for HRV parameters(N = 194), CRP(N = 100) and whole blood counts(N = 104) after adjusting for circadian variation, BMI and age.

^d^Comparison between baseline and next-morning using linear mixed regression models HRV parameters(N = 187), CRP(N = 94) and whole blood counts(N = 98) after adjusting for circadian variation, BMI and age.

Levels of WBC, platelets and RBC were significantly increased immediately after SHS exposure (WBC: 6.80 k/μl vs. 7.33 k/μl; Platelets: 216.8 k/μl vs. 221.5 k/μl; RBC: 4.91 M/μl vs. 4.83 M/μl), but no differences were observed the next morning (Table 3). As compared to baseline, CRP levels showed no difference post SHS exposure, but were higher the next morning, suggesting a delayed response. We also observed significant higher neutrophils both immediately following SHS exposure and the next morning. The levels of eosinophils, lymphocytes, basophils and monocytes showed no statistically significant differences between the three measurement periods.

We constructed linear mixed regression models to investigate the exposure-related relationship between HRV/inflammation and SHS, as measured by PM_2.5_. The regression coefficients for the PM_2.5_ models are summarized in Table 4. We found elevated PM_2.5_ concentrations were associated with lower levels of all post-shift HRV parameters- SDNN, rMSSD, LF and HF. Increased PM_2.5_ concentrations were also associated with higher post WBC and the next-morning CRP levels. No significant associations were observed between PM_2.5_ and other markers of inflammation.

**Table 4:** **Association between cardiovascular endpoints and PM**_**2.5 **_**exposure**^**a**^

**Variables**	**Post**		**Next morning**	
	**β Coefficient (SE)**	***p *** **Value**	**β Coefficient (SE)**	***p *** **Value**
^b^Heart rate(bpm)	2.9(0.5)	0.241	−0.3(0.7)	0.689
^b^SDNN(msec)	−7.8(3.0)	0.012	2.5(2.9)	0.389
^b^rMSSD(msec)	−8.0(3.9)	0.048	1.7(2.6)	0.516
^b^LF(msec^2^)	−17.2(6.3)	0.010	4.9(6.3)	0.441
^b^HF(msec^2^)	−29.0(10.1)	0.006	3.8(6.0)	0.525
^b^CRP(mg/L)	8.1(9.3)	0.445	24.2(9.0)	0.043
^b^Lymphocytes(k/μl)	1.7(2.1)	0.501	−3.1(2.6)	0.353
^c^Red blood cells(M/μl)	−0.004(0.017)	0.852	−0.011(0.025)	0.673
^c^White blood cells(k/μl)	0.424(0.144)	0.021	0.200(0.203)	0.356
^c^Neutrophils(k/μl)	−0.038(0.11)	0.756	0.076(0.118)	0.556
^c^Monocytes(k/μl)	−0.012(0.022)	0.655	−0.027(0.018)	0.211
^c^Basophils(k/dl)	0.001(0.001)	0.521	−0.003(0.016)	0.862
^c^Eosinophils(k/μl)	0.018(0.006)	0.106	−0.004(0.012)	0.737
^c^Platelets(k/μl)	−0.808(2.72)	0.778	−2.27(2.53)	0.404

^a^Linear mixed effects regression models were used to investigate the exposure-related changes in HRV(N = 97), CRP(N = 50) and whole blood cell count(N = 52). All models were adjusted for BMI, age, and circadian variation.

^b^Variables were log-transformed due to the skewed distribution of model residuals. Associations were calculated by the equation percentage (%) = [exp(beta coefficients)-1]*100 and are interpreted as percent change.

^c^Variables are untransformed and beta coefficients are presented from mixed regression models.

After excluding 2 participants taking statin or beta-blockers, the differences in HRV and inflammatory markers across the three time periods persisted (data not shown). The results of the PM_2.5_ exposure-response models showed slightly larger associations between PM_2.5_ and post WBC and next morning CRP; yet slightly smaller regression coefficients for post SDNN, rMSSD, LF and HF (Table 5).

**Table 5:** **Association between cardiovascular endpoints and PM**_**2.5 **_**exposure in sensitivity analysis**^**a,b**^

**Variables**	**Post**		**Next morning**	
	**β Coefficient (SE)**	***p *** **Value**	**β Coefficient (SE)**	***p *** **Value**
^c^Heart rate(bpm)	3.1(0.5)	0.239	−0.4(0.7)	0.680
^c^SDNN(msec)	−8.1(3.0)	0.010	2.7(2.9)	0.361
^c^rMSSD(msec)	−8.2(3.9)	0.001	1.7(2.7)	0.538
^c^LF(msec^2^)	−18.0(63)	<0.001	4.7(6.4)	0.471
^c^HF(msec^2^)	−29.3(10.2)	0.006	3.7(6.1)	0.551
^c^CRP(mg/L)	8.3(9.9)	0.464	25.7(9.3)	0.403
^c^Lymphocytes(k/μl)	1.7(2.2)	0.520	−4.5(2.8)	0.179
^d^Red blood cells(M/μl)	−0.001(0.018)	0.946	−0.015(0.026)	0.575
^d^White blood cells(k/μl)	0.433(0.146)	0.208	0.177(0.210)	0.426
^d^Neutrophils(k/μl)	−0.041(0.108)	0.744	0.058(0.122)	0.661
^d^Monocytes(k/μl)	−0.013(0.023)	0.630	−0.003(0.184)	0.145
^d^Basophils(k/dl)	0.001(0.001)	0.506	−0.053(0.165)	0.762
^d^Eosinophils(k/μl)	0.018(0.006)	0.115	−0.004(0.012)	0.740
^d^Platelets(k/μl)	−0.639(2.853)	0.830	−3.07(2.65)	0.291

^a^Linear mixed effects regression models were used to investigate the exposure-related changes in HRV(N = 94), CRP(N = 47) and whole blood cell count(N = 49). All models were adjusted for BMI, age, and circadian variation.

^b^Two participants who reported taking statin or beta-blockers were excluded in sensitivity analysis.

^c^Variables were log-transformed due to the skewed distribution of model residuals. Associations were calculated by the equation percentage(%) = [exp(beta coefficients)-1]*100 and are interpreted as percent change.

^d^Variables are untransformed and beta coefficients are presented from mixed regression models.

## Discussion

In the present study, we observed an immediate cardiovascular autonomic response, as measured by HRV, and both an immediate and delayed inflammatory response to short-term SHS exposure among non-smoking boilermaker construction workers. Immediately following acute SHS exposure we observed statistically significant decreases in the multiple 5-minute resting HRV parameters. The coefficients of the mixed linear regression model suggested that a 100 μg/m^3^ increase in PM_2.5_ was associated with 7.8% lower 5-minute SDNN and 17.2% lower 5-minute LF following SHS exposure. Though we did not observe significant changes in rMSSD and HF compared with baseline, there were inverse exposure-dependent relationships between these parameters (8% lower rMSSD and 29% lower HF) with a 100 μg/m^3^ increase in PM_2.5_. We did not find differences in the next-morning HRV parameters compared with baseline. Therefore, the results of this study suggest non-smokers experience rapid HRV response to acute SHS exposure that do not persist 18-hours following the end of exposure.

Our observations are consistent with the findings from a study conducted by Pope et al. [11] at an airport in Salt Lake City. In this study, participants were monitored with ECGs alternated between the environmental tobacco smoke (ETS) area and smoke-free area for 2 hours periods over an 8-hour observation window. Pope et al. observed negative associations between environmental tobacco smoke (ETS) and SDNN with about 12% decrements on average. A recent study on ETS and HRV was conducted by Wilson et al. [24] among 14 restaurant or bar workers. In this study, SDNN decreased 2.7% and rMSSD decreased 3.8% per mg-hr/m^3^ ETS exposure. A cross-sectional study conducted in the Swiss adult reported that exposure to ETS more than 2 hours/day was associated with significant decreases in LF, LF/HF and increases in heart rate [25]. While the magnitudes of changes in HRV from these studies are difficult to compare with due to different study designs, protocols for ECG monitoring and exposure assessment, findings from the different studies consistently show that SHS is associated with declines in HRV.

Previous studies have shown that secondhand smoke may induce adverse cardiovascular effects by disrupting functions of autonomic nervous system which is often captured by imbalance between sympathetic control and parasympathetic control [26]. LF and SDNN are often considered to be influenced by both the sympathetic nervous system (SNS) and parasympathetic nervous system (PNS), while HF and rMSSD are influenced by the PNS [27]. In our study, reduced SDNN and LF were observed following SHS exposure, yet no statistically significant changes were observed for rMSSD and SDNN. However, when we accounted for the quantity of exposure, evaluating the relationship PM_2.5_ and the HRV markers, we observed statistically significant associations with all four HRV parameters (SDNN, rMSSD, LF and HF). The results may suggest that SNS plays an important role in the altered cardiovascular response to SHS, yet does not preclude the involvement of the PNS.

We also found statistically significant positive exposure-dependent relationships between acute SHS exposure and inflammatory markers. Immediately following SHS exposure, a 100 μg/m^3^ increase in PM_2.5_ was associated with 0.42 k/μl higher WBC count. In addition, the next-morning CRP levels associated with a 100 μg/m^3^ increase in PM_2.5_ were 24.2% higher. Although there were significant higher platelets, RBC and neutrophils immediately after SHS exposure, these associations did not show a linear exposure-response relationship with PM_2.5_ concentrations. The sample size for participants who provided blood samples was small, which may have limited our ability to detect significant relationships. Another potential explanation is that the exposure-response relationship between inflammation and SHS exposure may be non-linear.

A study by Kim et al. [28] examined the acute inflammatory effects of welding fumes exposure among a cohort drawn from the same population of boilermaker welders. The study was conducted when the participants welded and were exposed to welding fumes for about 6 hours. The median PM_2.5_ concentration of the welding fumes was 1.66 mg/m^3^, which is nearly 12-fold more than the mean PM_2.5_ exposure in participants in our study. Kim et al. observed that acute welding fumes exposure was associated with increases in WBC counts immediately following welding fumes exposure and CRP levels 18-hrs following welding fume exposure. Among non-smokers, it was estimated that a 1 mg/m^3^ increase in PM_2.5_ was associated with 0.8 k/μl increases in WBC; the estimated increases in CRP associated with a 100 μg/m^3^ increase in PM_2.5_ were 0.9 mg/l in both non-smokers and smokers. The changes in WBC are approximately half the effects observed in the current study [0.4 k/μl per 100 μg/m^3^]. In an air pollution study, Pope et al. examined inflammatory effects of ambient PM_2.5_ exposure among 88 elderly subjects [5]. Pope et al. did not conduct personal PM_2.5_ monitoring but instead collected the daily data of PM_2.5_ concentrations during the winter/summer in three Utah communities. Pope et al. also observed significant increases in levels of CRP following PM_2.5_ exposures with a 100 μg/m^3^ increase in PM_2.5_ was associated with 0.81 mg/dl increases in CRP.

HRV and some markers of inflammation are known to exhibit a circadian pattern- the physiological and cyclical fluctuations in 24 hours [22,23]. For example, HRV parameters decreased from morning to night and then returned back in the next-morning [29]. In this study, the variable “time of baseline” that was used to indicate whether the baseline samples were taken in the morning or in the afternoon was controlled for in all linear mixed regression models. However this variable only accounted for differences in circadian variation between individuals at different measurement times, but not within individuals. Therefore, it is unclear whether the declines in SDNN, LF and white blood cells immediately following SHS exposure were due to circadian variation or SHS exposure. But given that there were exposure-response relationships between these variables and PM_2.5_, it is likely that SHS was responsible for some of these changes.

Studies have shown that activities, such as talking, laughing and walking can affect HRV by changing respiratory frequency [30,31]. In this study, we took resting ECG recordings among participants when they were seated quietly for about 7 or 12 minutes, which means that potentially confounding effects by those activities were controlled for. In addition, the repeated measures design of this study allowed us to control for individual differences among participants, which helped increase the validity of our results. Compared with fixed-point monitoring, we were able to monitor and quantify personal specific PM_2.5_ exposure among participants and this should help increase the accuracy of personal PM_2.5_ exposures.

The study is limited by the small sample size and the possibility that the constituents of tobacco by-products besides PM_2.5_ also contributed to the effects we observe. Among thousands of components in SHS, chemicals including carbon monoxide and nicotine are potential triggers of cardiovascular events. Those two chemicals, however, were unlikely to have a major effect on cardiovascular effects of SHS as Pope et al. [11] observed consistent decline in HRV among non-smokers that were exposed to SHS though the levels of carbon monoxide were extremely low. In a study investigating the cardiovascular automatic response of nicotine patch administration, Lucini et al. observed very small changes in HRV parameters though the nicotine levels were high [32]. Although ambient ozone [33,34] and nitrogen dioxide [35] were reported to be associated with lower HRV in many air pollution studies, they were unlikely to confound the cardiovascular effects by SHS since the participants stayed in welding room and no welding occurred during the day.

In the present study, we did not conduct PM_2.5_ monitoring during the night of the SHS exposure day due to the inconvenience for participants. However, it could be possible that some of participants were exposed to ambient air pollutants or SHS at home, which may partially contribute to the changes in the next-morning CRP levels. We did not monitor air pollution levels in the welding room and it is likely that seasonal PM_2.5_ may change. However, Ren et al. [36] monitored daily average PM_2.5_ exposure among 320 men who lived in greater Boston area and reported the daily average was 13.0 μg/m^3^, which is nearly one tenth of the 6-hour average PM_2.5_ exposure (132.3 μg/m^3^) in our study. Therefore, the background PM_2.5_ may contribute to minor cardiovascular effects in the present study. We only evaluated the cardiovascular effects of PM_2.5_, but future studies should examine the components of the PM_2.5_ and ultrafine particles, which may be particularly hazardous [37]. Furthermore, future studies may investigate endothelial dysfunction, increased blood pressure and coagulation effects [38], which are other pathways that have been suggested to account for the cardiovascular responses to short-term SHS exposure.

## Conclusions

In this study, we hypothesized that altered cardiovascular automatic response and inflammatory response as seen by decreases in HRV and increases in inflammatory markers levels would be associated with acute SHS exposure, as quantified by PM_2.5_ concentrations, among non-smoking boilermaker construction workers. We observed lower exposure-related levels of HRV immediately following exposure while higher levels of CRP were not observed until 18 hours following exposure. The results support the hypothesis that cardiovascular autonomic and inflammatory responses may be on the pathophysiologic pathways that link SHS exposure and cardiovascular disease.

## Abbreviations

SHS: Secondhand smoke; CVD: Cardiovascular disease; ECG: Electrocardiogram; HR: Heart rate; HRV: Hear rate variability; CRP: C-reactive protein; WBC: White blood cell count; SDNN: The standard deviation of NN intervals; rMSSD: The square root of the mean of the sum of the squares of the successive differences between adjacent NNs; LF: Power in the low frequency range (0.04-0.15 Hz); HF: Power in the high frequency range (0.15-0.4 Hz); SNS: Sympathetic nervous system; PNS: Parasympathetic nervous system; SD: Standard deviation; SE: Standard error.

## Competing interests

The authors declare that they have no competing interests.

## Authors’ contributions

JZ contributed to data collection, statistical analysis and interpretation of the results and drafted the manuscript. FCS contributed to data collection, interpretation of the results and review of the manuscript. MAM performed ECGs analysis and contributed to review of the manuscript. DCC contributed to the study design, data collection and review of the manuscript. JMC contributed to the study design, data collection and exposure assessment, statistical analysis and review of the manuscript. All authors read and approved the final manuscript.
